# Optic Pathway Gliomas in Pediatric Population—Current Approach in Diagnosis and Management: Literature Review

**DOI:** 10.3390/jcm12216709

**Published:** 2023-10-24

**Authors:** Monika Modrzejewska, Joanna Olejnik-Wojciechowska, Agnieszka Roszyk, Elwira Szychot, Tomasz Dariusz Konczak, Marcin Szemitko, Jarosław Władysław Peregud-Pogorzelski

**Affiliations:** 1 II Department of Ophthalmology, Pomeranian Medical University, Al. Powstańców Wlkp. 72, 70-111 Szczecin, Poland; 2 Scientific Students Association of Ophtalmology, II Department of Ophthalmology, Pomeranian Medical University, Szczecin Unia Lubelska 1 Street, 71-252 Szczecin, Poland; 3 Department of Paediatrics, Oncology and Paediatric Immunology, Pomeranian Medical University, 71-252 Szczecin, Poland; 4 Department of Paediatric Onclogy, Great Ormond Street Hospital for Children, London WC1N 1LE, UK; 5 Department of Intervantional Radiology, Pomerian Medical University, 70-111 Szczecin, Poland

**Keywords:** optic glioma, OCT, VA, treatment management, biopsy, molecular therapy, NF-1, *BRAF*-mutation, histopathological types, pilocytic astrocytoma

## Abstract

In this paper, the authors present a clinical picture of the diagnosis and current treatment regimens of optic pathway glioma in the pediatric population, with an emphasis on the role of an ophthalmic diagnosis in the differentiation and monitoring of lesions. Glioma is the most common optic nerve tumor in children. Material: Articles in PubMed, Scholar and Website were reviewed, taking into account current standards of management related to sporadic or NF1-related optic glioma, epidemiology, location, course of the disease, clinical manifestations, histological types of the tumor, genetic predisposition, diagnostic ophthalmic tests currently applicable in therapeutic monitoring of the tumor, neurological diagnosis, therapeutic management and prognosis. The importance of current screening recommendations, in line with standards, was emphasized. Results: Glioma occurs in children most often in the first decade of life. Initially, they may be asymptomatic, and clinically ophthalmic changes are associated with the organ of vision or with systemic changes. Gliomas associated with the *NF1* mutation have a better prognosis for sporadic gliomas. Diagnosis includes radiological imaging methods/MRI/ophthalmology/OCT and visual acuity log MAR assessment. The basis of treatment is clinical observation. In the case of disease progression, surgical treatment, chemotherapy and targeted therapy are used. Conclusion: Further research into novel techniques for detecting gliomas would allow for early monitoring of the disease.

## 1. Introduction

Gliomas are a type of brain tumor that develops from neuroglial progenitor cells. [1] Gliomas arising from the optic pathway represent approximately 2–5% of intracranial tumors in children [2,3,4,5,6,7,8,9,10]. These tumors affect the pre-cortical visual pathway and can grow anywhere along this pathway, extending over the optic nerves up to the occipital lobe cortex area, and involve both the area of the hypothalamus and the optic chiasm, which are essential for binocular vision [11,12]. OPGs can be divided into those that are sporadic and those associated with neurofibromatosis type 1 (NF1) [13,14,15,16,17]. The tumor arises in approximately 15 to 30% of children with NF1, a tumor predisposition syndrome caused by the germline mutation in NF1 gene. Mutations of *NF1*, located on chromosome 17q, are linked to the loss of neurofibromin, resulting in the activation of the RAS signaling pathway [18]. OPGs occur in the first decade of life, and the incidence decreases with age [7,19]. Survival outcomes of pediatric patients with OPGs are generally good, with 20-year overall survival rates between 87% and 91% [20,21,22,23,24]. Sporadic OPGs also have identifiable genetic factors that contribute to their formation. The most common somatic genetic alteration is a genomic rearrangement that results in a fusion transcript in which the kinase domain of the BRAF gene is fused to a gene of unknown function (*KIAA1549*) [13,16,25]. The two most commonly described aberrations are an activating *BRAFv600E* point mutation and a tandem duplication resulting in a *KIAA1549-BRAF* fusion. This fusion results in a transcript in which the kinase domain of the BRAF gene is fused to a gene of unknown function (*KIAA1549*) [26], leading to increased BRAF activation of the MEK signaling cascade. In addition, other potential driver mutations or fusions include the KRAS, FGFR1, PTPN11, RAF1 and NTRK2 genes [27,28,29,30]. The *BRAF v600e* mutation may be protective [31]; other genetic alterations of the RAS-MAPK pathway are often described in sporadic OPG cases [16,25,29,30,32,33,34]. The most common histologic type of optic nerve gliomas is pilocytic astrocytoma, accounting for 50% to 73% of all primary optic nerve tumors and 1.5 to 4% of all orbital tumors [18,35,36,37]. 

## 2. Location

Pilocytic astrocytoma (PCA) is the most common childhood brain tumor [38,39] and tends to occur close to the midline of the brain. It most commonly occurs in the posterior fossa. In 42–60% of cases, it occurs in the cerebellum, while 9% to 30% cause gliomas of the optic nerve and hypothalamus, as well as those associated with *NF1* mutations [39,40].

In contrast, the sporadic form is most often located behind the optic nerve junction, mainly in the cerebellum [13,41]. The location of optic nerve gliomas is divided into prechiasmatic, chiasmatic or postchiasmatic. Optic nerve glioma usually spreads along the optic nerve, and distant metastasis is rarely described [12]. In a study led by Azzizi and Walker [19], 83 patients with *NF1* mutation were analyzed. The study showed that 37% of tumors were located behind the optic chiasm, and 76% of gliomas occurred bilaterally. 

## 3. Epidemiology

Gliomas of the optic nerve are far more common in the pediatric population. According to the available literature, gliomas develop in 75% of patients in the first decade of life, and in 90% in the first two decades of life [8]. Gliomas of the optic nerve and optic chiasm are equally common in both sexes. However, the incidence is higher among females (65%) compared to males (35%) for tumors confined to the optic nerve only [34]. Approximately 15–20% of patients with NF1 will eventually develop OPG, but only 30–50% of them will develop clinical symptoms [13,42]. An analysis of 1257 OPGs cases showed that people from the Caucasian population were most likely to be diagnosed with this tumor type. These findings were also confirmed by Peckham-Gregory et al. in an analysis of 709 cases [37,43]. 

## 4. Course

OPGs are slow-growing tumors. They can present with accelerated and stunted growth, as well as spontaneous regression. These tumors usually spread along the optic nerve, and distant metastasis are rarely described [12,23,37,43]. Patients with coexisting NF1 have a less dynamic course and less often require treatment than patients with sporadic gliomas, where the progression often requires several therapeutic approaches [35,44,45,46]. NF1-associated gliomas involve the optic nerve, optic chiasm and/or optic radiations. Sporadic known *BRAFv600E* mutations are larger and have a tendency toward infiltration, greater progression and tumor recurrence [47,48,49]. An important aspect of the approach in patients with OPGs is often the inability to perform a biopsy, which results in the lack of histopathological and, especially, molecular diagnosis. Patients with a detected *BRAF V600E* mutation in the pediatric group show positive overall survival (OS) outcomes, while such a correlation is not confirmed in the adult population [50]. The prognosis of OPGs is also influenced by the therapeutic modalities used for treatment. Radiotherapy is not recommended for children with NF1-associated OPGs, because of the risk of secondary malignant tumor development within radiation fields [51]. 

## 5. Histological Type

The most common histologic types found in children with OPGs are low-grade gliomas (WHO grade I and II), with the most common being pilocytic astrocytoma (PCA) and the rarer pleomorphic xanthoastrocytoma (PXA). We include pilocytic astrocytoma, subependymal giant cell astrocytoma (SEGA), diffuse astrocytoma, pilomyxoid astrocytoma and Pleomorphic xanthoastrocytoma in the group of tumors classified as astrocytic tumors within the low-grade glioma (LGG) category [5,18,35,36,37,41,52]. Histological features such as atypia, anaplasia, microscopic proliferation and necrosis play a crucial role in distinguishing between low-grade and high-grade tumors. Low-grade gliomas (LGGs) are characterized by well-differentiated and densely packed glial cells with nuclear atypia and limited mitotic activity. Additionally, the specific appearance of cells, like the “fried egg” appearance in oligodendrogliomas and the presence of pleomorphic giant cells in astrocytomas, allows for the differentiation of these tumor types. As a result, histological classification is feasible for these tumors [51]. Sporadic gliomas of the visual pathway (not associated with the *NF1* mutation) show more aggressive histopathological features compared to gliomas associated with this mutation [7,13,35,47].

## 6. Genetic Predisposition

As previously mentioned, OPGs can either occur sporadically [50] or be associated with a genetic predisposition. OPGs among patients with a genetic predisposition typically occur earlier on in life, at around 4–5 years of age on average, and can be both unilateral and bilateral [3,44,46]. Pilocytic astrocytoma (PCA) of the visual pathways developing in the course of NF1 have been described in some articles as being more common in the female gender [46,53]. However, other articles show no such relationship [45,54,55,56]. No association has been discovered between the incidence of this cancer and the patient’s race [57].

*NF1* is a tumor suppressor gene that controls cell proliferation, survival and differentiation, and it is inactivated in Recklinghausen disease. The mechanism works through the mitogen-activated kinase (MEK) and mTOR pathways [41,58]. The product of the NF1 gene (neurofibromin 1) normally binds to RAS, thereby inactivating it and controlling cell division. Consequently, a lack of NF1 gene activity results in constitutive activation of RAS and uncontrolled cellular proliferation [3]. In patients with NF1, inactivation of the NF1 gene leads to increased RAS activity and subsequent activation of the MAPK pathway. All of these observations point to the central role of abnormal MAPK pathway activation as the most common genetic aberration detected in children with LGG, both in relation to clinical course and PCA biology [14,15,16,17]. 

NF1-associated OPGs contain a bi-allelic inactivation of the NF1 tumor suppressor gene (neurofibromin 1; MIM 613113). Consequently, patients with NF1 are born with one mutated copy of the NF1 gene, and tumors develop after acquired loss of the remaining *NF1* in the tumor without any other recurrent oncogenic alterations [32,33]. The clinical diagnosis of NF1, in addition to the ocular manifestations in the case of gliomas, is based on clinical diagnostic criteria. The main features of NF1 are café-au-lait (CAL) spots, axillary or inguinal/perineal freckling, neurofibromas, Lisch nodules, distinctive osseous lesions, predisposition to the development of benign and malignant tumors of the nervous system [52]. 

## 7. Symptoms

The symptoms of OPGs depend primarily on their location: in the orbit or in the CNS. For orbital tumors, the most common symptom is exophthalmos, often with strabismus (94%) and early vision loss (88%). Other common symptoms include abnormal pupillary response, dyschromatopsia, optic atrophy, swelling of the optic disc, nystagmus and elevated intraocular pressure [8,18]. Patients may present with hydrocephalus (27%) or endocrine disorders (26%) if the tumor is located in the region of the optic chiasm, especially with hypothalamic involvement [36]. The clinical symptoms of hypothalamic tumors can lead to pituitary insufficiency and, consequently, endocrine insufficiency in multiple systems, such as secondary thyroid insufficiency and adrenal insufficiency. Additionally, disturbances in sexual maturation and growth deficiency can be observed. For some children, the initial symptoms may include signs of increased intracranial pressure, such as nausea, vomiting, headache, bradycardia or hypertension [59]. Some OPGs, especially those coexisting with NF1, remain asymptomatic and may be detected incidentally during screening [46,53]. The presence of *NF1* mutations is also important in the dynamics of symptoms—sporadic optic nerve gliomas are generally detected in the first decade of life and carry a higher risk of vision loss [3]. In contrast, more than 50% of NF1-related OPGs are not associated with visual impairment [60]. An analysis of 83 patients with OPGs showed that [61] 52% of patients were asymptomatic, 30% had only one ocular symptom and 18% had multiple ocular symptoms. Frequent ophthalmic clinical signs and symptoms were present in more than 10% of patients and included strabismus (22/83, 27%), ocular motility disorders and exophthalmos (11/83, 13%) [61]. Clinical manifestations of hypothalamic tumors can lead to premature or delayed sexual maturation by affecting the inhibition of gonadotropin secretion; growth hormone deficiency leads to growth retardation, and thyroid dysfunction results in TSH deficiency and steroid axis disorders with ACTH deficiency. Additional symptoms include seizures, nausea, dizziness and developmental regression [2,18,62]. According to a study by Heidary, Fisher et al. [63], glioma localized in the visual pathways and/or visual radiations is a major risk factor for visual field deficits at the end of treatment. 

## 8. Neurological and Ophthalmic Imaging Diagnostics

The key diagnostic investigations of OPGs include a neurological clinical examination, visual acuity (VA), assessment by logMAR arrays and OCT (optical coherence tomography), and magnetic resonance imaging (MRI). It is important to correlate the symptoms presented with supplementary gadolinium-enhanced MRI images. MRI provides a distinct advantage over CT [6,12,64]. MRI can detect tumor expansion beyond the optic nerve into the optic chiasm, which is not visible on CT. When administering a gadolinium-based contrast agent, some ONGs can obtain high enhancement, which may speak to their high metabolic activity [65]. New MRI techniques combined with OCT are helping to identify preclinical signs of neuronal loss and, thus, those that are more vulnerable to compression. It is, therefore, important that eligibility criteria for future studies include evidence of prior visual deterioration (if such evidence can be found) as a factor in case selection for treatment and in the analysis of visual effects. Optic nerve atrophy is examined by OCT, which is a noninvasive technique that assesses retinal fiber layer (RNFL) thickness, which correlates with neuronal loss, demonstrating an objective assessment of visual pathway damage, with early attempts at therapeutic intervention [66,67,68]. The OCT examination should be performed with the pupil dilated, after the visual acuity assessment, but before the optic disc examination. This order increases the reliability of the examination [69,70]. The RNFL thickness has been shown to be a marker of visual pathway damage [71]. The RNFL value correlates with VA values. It has higher sensitivity and specificity than VA and ophthalmoscopic evaluation of the optic disc. Studies indicate that OPGs cause loss of RNFL before the onset of clinical symptoms, which would confirm the great importance of OCT in the early detection of optic nerve damage [70,72]. Due to its duration and accuracy of measurement, it is a test performed successfully even in the youngest patients. Therefore, it is applicable in clinical practice [65,70]. It has been proven that a decrease in RNFL values of more than 10% correlates with an increase in visual loss. In turn, constant RNFL values in subsequent studies are predictive of the stabilization of visual acuity [73]. Spectral-domain optical coherence tomography (SD OCT) with near-infrared (NIR) reflection is able to detect choroidal nodules, which are characteristic of OPGs associated with NF1 (NF1-OPG). SD-OCT with NIR is currently being evaluated as a new diagnostic criterion in patients with NF1 [74,75]. A new method under evaluation is OCT angiography. This is a noninvasive test that does not require dye administration; it allows for a precise assessment of the vascularization of the retina and optic nerve disc [68,76]. An additional method to facilitate diagnosis may be automated perimetry, which can visualize damage to the optic nerve and optic chiasm more quickly and accurately than visual acuity testing; however, this assessment may be more difficult in children [6,11]. 

According to an analysis by Heidary et al. [63], at the end of treatment, visual field defects were present in 76% of patients (19/25 children), which may indicate that children with symptomatic NF1-OPG have a high prevalence of visual field defects. It is worth noting that these findings underscore the need to include visual field assessment as a standard part of clinical care both at presentation and throughout the clinical course of OPG treatment. Biopsy, if feasible, is also an important diagnostic component that is performed to select the most appropriate targeted therapy, especially in OPGs following multiple lines of chemotherapy. 

Laboratory investigations should include tumor markers (AFP and B-HCG) for suprasellar lesions detected on imaging to exclude germ cell tumors [77,78]. Currently, with the use of molecular diagnosis employing Next-Generation Sequencing (NGS) methods, we can identify known mutations within the BRAF and NF1 genes. In cases where the clinical presentation is clear but the NGS test is negative, one may consider expanding the diagnosis with Whole-Exome Sequencing (WES). It is important to note that both somatic and germline cells should be evaluated [79,80]. The course of gliomas is unpredictable in some cases and can be rapid. It is important to note that a thorough knowledge and understanding of the molecular alterations in the glioma by biopsy will allow for a better therapeutic outcome [81].

## 9. Current Recommendations for Screening

Recommendations for screening patients with optic nerve glioma in NF1 include annual testing of visual acuity on logMAR boards in all children under the age of 8 and at least every 2 years until the age of 18 [42]. A meta-analysis by de Blank et al. reports that once the diagnosis is established, most centers perform VA testing every 3 months for the first year, then every 6 months for 2 years until age 8 and then annually until age 18 [82]. Visual acuity testing in patients with NF1 is most critical for monitoring treatment and should be performed by an experienced ophthalmologist. According to recent guidelines, visual acuity assessment plays the most important role in assessing disease progression and in making therapeutic decisions. Therefore, regular visual acuity testing is recommended for patients with OPGs [72,82]. Visual acuity is assessed using Lea symbols, Snellen tables and logMAR. [68,70,83]. Continuous follow-up with regular visual acuity assessments and MRI surveillance scans allows for the evaluation of the disease and its progression. It should be noted, however, that disease progression during surveillance is not strictly correlated with changes in the tumor volume [84]. If progression in terms of decreased visual acuity is detected, other causes, such as refractive defects or lack of patient cooperation, should be ruled out first before the ophthalmic test results can be linked to the OPG’s progression. In addition, visual acuity testing should be repeated in 1–2 weeks [85]. Patients with NF1 may have difficulty concentrating and impaired cognitive function, and children 5 years old and younger are also unable to proceed and complete the visual acuity test. Equivalent tests have been created, but unfortunately, none of them can be considered a substitute for visual acuity testing, and they cannot be used to decide whether to implement treatment [70]. Visual field testing is a test that has been proposed as a supplementary test for visual acuity. Unfortunately, the results of this examination depend on the patient’s cooperation, concentration and patience. It is not recommended to perform this test in patients younger than 7 years of age [70,72,82], as included in the reference recommendations of authors Patel, Cumberland et al. [86]. Visual evoked potentials (VEPs) were proposed as a screening test by de Blank, Fisher et al. [82]. Unfortunately, due to its high sensitivity but low specificity, as well as the long time required to perform the test, which is problematic in young children, the study of visual evoked potentials has not found application in clinical practice [70,72,87]. The examination and evaluation of eye movements should also be mentioned. If strabismus and nystagmus are associated with OPG, they are often accompanied by loss of visual acuity [2,13]. Assessment of the optic nerve disc should also be performed. It is necessary to seek for signs of obliterated borders or features of swelling of the optic disc. However, it is important to remember that edema does not always correlate with VA loss. The decision on progression and treatment should not be made based on this examination alone [72,85]. 

## 10. Current Guidelines for the Diagnostic Procedure in Patients with Optic Nerve Glioma

According to the current guidelines, in the observation group without residual tumor or with stable residue, physical and neurologic examination and ophthalmology (mandatory in VPG) should be performed every 3 months, audiometry (for brain stem LGG) should be performed every 6 months, and MRI should be performed every 3–6 months during the first year. In the second year, physical and neurologic examination, MRI and ophthalmology should be performed every 3–6 months. 

In the 3rd–5th years, physical and neurologic examination should be performed every 6 months, while MRI and ophthalmology (mandatory in VPG) should be performed every 6–12 months. 

In 6th–10th years, physical and neurologic examination should be performed annually, and MRI should also be performed annually, although optionally every 6 months in the case of VPG. Ophthalmology (mandatory in VPG) should be performed every 6–12 months. 

In the 1st–10th years, neuro-cognitive follow-up is according to institutional policy. In the 2nd–10th years, audiometry is not indicated if the results were previously normal, but there should be regular assessments if hearing is impaired. 

In the chemotherapy group with stable residue after termination of treatment, physical and neurologic examination with auxology, MRI (cranial and/or spinal) and ophthalmology (mandatory in VPG) should be commissioned every 3 months; audiometry (following carboplatin and for brain stem LGG) and assessment of renal function should be performed 6 months after chemotherapy.

In the second year, physical and neurologic examination, MRI and ophthalmology should be performed every 3–6 months. In the 3rd–5th years, physical and neurologic examination, MRI and ophthalmology should be performed every 6 months. In 6th–10th years, physical and neurologic examination should be performed annually, and MRI should also be performed annually, although every 6 months in the case of VPG. Ophthalmology (mandatory in VPG) should be performed every 6–12 months. In the 1st–10th years, neuro-cognitive follow-up is according to institutional policy.

In the 2nd–10th years, audiometry is not indicated if the results were previously normal, but there should be regular assessments if hearing is impaired. Renal function should be performed yearly, if not otherwise indicated; this assessment may not be required if the results are repetitively normal.

In the radiotherapy group with stable residue after termination of treatment, physical and neurologic examination with auxology, MRI (cranial and/or spinal) and ophthalmology (mandatory in VPG) should be commissioned every 3 months, while audiometry (following irradiation of the inner ear and for brain stem LGG) should be performed every 6 months. 

In the second year, physical and neurologic examination, MRI and ophthalmology should be performed every 3–6 months, and audiometry (following irradiation of the inner ear and for brain stem LGG) should be performed every 6–12 months. In the 3rd–5th years, physical and neurologic examination with auxology should be performed every 6 months, MRI and ophthalmology (mandatory in VPG) should be performed every 6–12 months, and audiometry (following irradiation of the inner ear and for brain stem LGG) should be performed annually. 

In the 6th-10th years, physical and neurologic examination should be performed annually, and MRI should also be performed annually, although every 6 months in the case of VPG. Ophthalmology (mandatory in VPG) should be performed every 6–12 months. Audiometry is not indicated if the results were previously normal, but there should be regular assessments if hearing is impaired.

In the 1st–10th years, neuro-cognitive follow-up is according to institutional policy [88].

## 11. Treatment

The SIOP-E-BTG and GPOH Guidelines for Diagnosis and Treatment of Children and Adolescents with Low-Grade Glioma include recommendations for the management of patients with OPGs. The management approach depends on the patient’s age, tumor location, histopathological, especially molecular diagnosis, associated cancer predisposition syndrome and other co-morbidities carrying a mutation in the *NF1/BRAF* gene. The main goal of therapy is directed at reducing the risk of permanent, clinically significant visual dysfunction. In clinical practice, observation is the most common approach. The decision to treat or observe may not be straightforward. The best indication for treatment with chemotherapy is evidence of progressive, clinically significant vision loss. There are currently two distinct criteria for transitioning from observation to treatment. The first criterion includes patients diagnosed with significant symptoms, such as diencephalic syndrome, and all infants under the age of 1 diagnosed with chiasmatic/hypothalamic glioma with remaining tumors, regardless of symptoms. The second criterion encompasses cases where there is substantial radiological progression of a residual tumor or the appearance of new lesions on MRI imaging that cannot be addressed surgically, as well as significant worsening of neurological or visual symptoms after the initial observation period. In the case of surgical treatment, the method of choice is complete resection while maintaining a microscopic disease-free margin. Due to the risk of vision loss, often, full radicality of the procedure may not be possible. This creates a significant clinical dilemma; whether a patient should be included in treatment or remain under observation after a partial resection is a crucial decision. Performing surgery in OPGs provides valuable information for both histopathological and molecular diagnoses [88]. 

Surgical treatment is not recommended upfront, but according to the literature, it may be considered when there are symptoms such as tumor pressure on the visual pathway, causing severe visual disturbances; pain; ocular exophthalmos; and infiltration of the hypothalamus by the glioma, causing endocrine disruption and affecting surrounding structures [2,5,89]. The most common surgical method is lateral transorbital access with preservation of the eyeball, along with the extraocular muscles, if possible [18]. In the case of surgical treatment, we encounter another problem, which is the resection of the tumor with margins. In the groups of patients studied, resection of the tumor with or without a margin was not noted to have favorable results [7]. Drug treatment of OPGs has been used for four decades. The results of chemotherapy trials are consistent in showing very good overall survival of between 70 and 95% but a low progression free survival, at around 45%, at 5 years. The standard chemotherapy combination remains vincristine and carboplatin (VCR/CBDCA) in both Europe and in the USA; however, the schedule and doses of the two drugs are not identical. 

Second-line chemotherapy may be required in the face of a hypersensitivity reaction to carboplatin or upon progression during treatment or early relapse after the completion of first-line chemotherapy. The following are alternative therapies: vincristine/cisplatin/cyclophosphamide, monotherapy with vinblastine, irinotecan/bevacizumab, and thioguanine/procarbazine/cisplatin/vincristine (TPCV) [4,90,91,92]. Currently, monotherapy with vinblastine (VBL) is increasingly being used over VCR/CBDCA. This approach is associated with a lower frequency of adverse effects, such as neutropenia, anemia, and thrombocytopenia, but it also carries a significant neurotoxicity risk to the autonomic nervous system. There are no definitive studies establishing the superiority of one therapy over the other, and within various publications, they can differ from one another. This should be addressed in the next SIOPE LGG trial. Research is ongoing on the vinblastine and bevacizumab protocol. While the results are not yet known, the initial information appears to be positive [93].

In gliomas, there is a significant increase in angiogenesis, with the growth factor VEGF inducing angiogenesis. Bevacizumab is a type of targeted therapy called an angiogenesis inhibitor. It is a monoclonal antibody that binds to a protein called VEGF, which is produced by some cancer cells and has been used in various pediatric tumors over the last 15 years with only limited benefit confined to selected groups, such as PLGG [94,95]. As a result of its action, tumor growth is reduced. Recently, bevacizumab has begun to be used with good effect in monotherapies and in combination with conventional drugs [93,96,97,98,99,100,101,102]. Unfortunately, bevacizumab is characterized by numerous side effects. The most common side effects of bevacizumab are hypertension, fatigue, joint pain, bleeding and proteinuria. These disappear after cessation of treatment [88,93,101,102]. Due to such beneficial results, further studies and research are required to select patients for inclusion of treatment at the appropriate time of disease progression [7,35,36,103,104]. 

Interesting research results came from a multicenter study presented by Green et al. about standardized neuro-radiological (RANO-LGG) and visual (logMAR visual acuity) criteria which were used to assess the clinical–radiological correlation and survival outcomes in examined group of patients. Bevacizumab provides effective short-term control of PLGG and delays further progression, providing better visual benefits than imaging methods. Bevacizumab is a safe and effective treatment for low-grade glioma in children, including the stabilization or improvement of visual acuity, which correlates best with MRI responses [104]. 

Although radiotherapy (RT) may provide favorable long-term outcomes in for either an adjuvant or salvage aim, chemotherapy is the preferred treatment approach due to the late effects of RT and impact on the optic pathway. RT induces apoptosis of local cells, and in the case of optic pathway gliomas (OPGs), it can lead to the worsening of vision. Proton beam radiotherapy may allow normal tissue sparing of radiation exposure compared to conventional photon therapy (references). Radiation therapy can be considered in cases where chemotherapy is not effective in the treatment of OPGs. However, the impact of radiation therapy, especially on patients with NF-1, should be taken into account, as it significantly increases the risk of secondary tumors. Therefore, it should not be the treatment of choice for NF-1 children with pediatric OPGs. In older children diagnosed with low-grade glioma outside of the optic pathway, radiotherapy may be considered as the first line of treatment [105].

As mentioned earlier, pilocytic astrocytoma is the most common type of childhood cancer. It is characterized by the presence of changes in the MAPK pathway. The mutation leads to the activation of and changes in cell modulation and proliferation. Mutation in the MAPK pathway makes it possible for MAPK inhibitor therapy to be used in the course of pilocytic astrocytoma. This allows for targeted therapy, which is very promising, but further research and analysis of the impact of this treatment on glioma is still ongoing [31,35,58]. Tovorafenib, a highly selective type II pan-RAF kinase inhibitor, was approved in the US this year [106,107]. 

The optimal duration of targeted therapy is still unknown. Therefore, in many cases, treatment is continued without interruption if it does not cause adverse effects. There are reports that the discontinuation of therapy can cause relapse or progression of the disease [36]. Currently, chemotherapy appears to be the first line of treatment. Despite side effects such as myelosuppression and risk of infection, among others, these are proven drugs that work well. Chemotherapy is a readily available treatment with specific criteria and regimens, making it easier to manage the patient. Targeted therapy is not yet thoroughly systematized. Toxicity effects such as skin lesions, cardiac dysfunction, infections, optic nerve neuropathy, uveitis and retinopathy, among others, are well-known. However, some side effects of such a treatment and its long-term effects remain unknown due to the complexity of cellular mechanisms and pathways [4,36].

## 12. Prognosis

Most gliomas are considered to have a low risk of malignant transformation [34,35]. The 20-year survival rate in children with OPG > 3 years of age is 91%, and the current concern is primarily the growing problem of long-term visual impairment [36]. However, the available literature manages to detail significant predictive factors for poor prognosis of visual pathway gliomas, such as an early age of onset, i.e., between 1 and 3 years; site of origin (postchiasmatic—behind the junction); and the presence of hypothalamic symptoms—along with increased intracranial pressure, partial resection and the absence of mutations in NF1. [5,31,62]. More than one clinical symptom, along with severe visual impairment, often occurred in patients who started treatment at a later stage, which was associated with a worse prognosis and was also associated with shorter PFS (progression-free survival) [10,31]. Typically, symptomatic cases occur in sporadic OPG rather than in NF1-OPG. In addition, NF1-associated OPG usually occurs before the junction, which is associated with a better prognosis. Tumors of the optic chiasm or behind the junction are associated with increased progression and mortality and are usually bilateral [13]. For both ophthalmologists and pediatricians, the most important thing is to periodically examine the search on the logMAR boards as soon as possible in order to detect a change in the progression of the disease.

## 13. Conclusions

 Due to the potential for a wide range of ophthalmic and general clinical symptoms in patients with optic pathway gliomas, multidisciplinary collaboration between ophthalmologists, oncologists, neurosurgeons, neuroradiologist, radiotherapists, endocrinologists, psychologists and geneticists, who have a significant impact on the early well-being of the patient and their caregivers, is extremely important. Isolated optic pathway gliomas are highly active tumors that tend to progress radiologically and cause visual deterioration in a significant percentage of patients compared to other OPG locations. Surgical treatment should be a last resort in the present day, and the main surgical intervention that is gaining in significance is biopsy, as it allows for the selection of appropriate targeted therapy, which currently remains in clinical trials. Chemotherapy deserves further pharmacological research, with perhaps new drugs, as it can help inhibit vision loss and is currently the most widely used and best-studied treatment, with the best long-term effects. The mainstay of OPG management today is observation and assessment of progression in correlation with clinical symptoms. Clinical signs and symptoms, optic nerve atrophy and history as predictive factors in children with NF1-OPG are of utmost importance. Therefore, eligibility criteria for future studies should include the timing of symptom onset, with a particular focus on visual deterioration, if identifiable. Further research into new techniques for the early detection of gliomas would allow for accurate monitoring of the disease, providing a chance for earlier treatment and longer survival for affected children. 

## Data Availability

Not applicable.

## Abbreviations

CNS	central nervous system
OPGs	optic pathway gliomas
NF1	neurofibromatosis type I
LGG	low-grade glioma
pLGG	pediatric low-grade glioma
PCA	pilocytic astrocytoma
PXA	(pleomorphic xanthoastrocytoma)

## References

[1] Xu S., Tang L., Li X., Fan F., Liu Z. (2020). Immunotherapy for Glioma: Current Management and Future Application. Cancer Lett..

[2] Avery R.A., Fisher M.J., Liu G.T. (2011). Optic Pathway Gliomas. J. Neuro-Ophthalmol..

[3] Beres S.J., Avery R.A. (2017). Optic Pathway Gliomas Secondary to Neurofibromatosis Type 1. Semin. Pediatr. Neurol..

[4] Farazdaghi M.K., Katowitz W.R., Avery R.A. (2019). Current Treatment of Optic Nerve Gliomas. Curr. Opin. Ophthalmol..

[5] Aihara Y., Chiba K., Eguchi S., Amano K., Kawamata T. (2018). Pediatric Optic Pathway/Hypothalamic Glioma. Neurol. Med.-Chir..

[6] Wladis E.J., Adamo M.A., Weintraub L. (2021). Optic Nerve Gliomas. J. Neurol. Surg. Part B Skull Base.

[7] Shofty B., Ben-Sira L., Kesler A., Jallo G., Groves M.L., Iyer R.R., Lassaletta A., Tabori U., Bouffet E., Thomale U.-W. (2017). Isolated Optic Nerve Gliomas: A Multicenter Historical Cohort Study. J. Neurosurg. Pediatr..

[8] Lin X., Huang R., Su J., Li H., Liu Z., Zhang P., Tian X. (2022). Optic Nerve Gliomas in Adults: A SEER-Based Study. All Life.

[9] Jahraus C.D., Tarbell N.J. (2006). Optic Pathway Gliomas. Pediatr. Blood Cancer.

[10] Jaing T.-H., Lin K.-L., Tsay P.-K., Hsueh C., Hung P.-C., Wu C.-T., Tseng C.-K. (2008). Treatment of Optic Pathway Hypothalamic Gliomas in Childhood: Experience with 18 Consecutive Cases. J. Pediatr. Hematol. Oncol..

[11] Pas C.B., Tanajura G.H., Giampani Junior J., de Brito A.G. (2022). Glioma of the Optic Nerve and Chiasm: A Case Report. Arq. Bras. Oftalmol..

[12] Krasodomska K., Lubiński W., Masztalewicz M., Cyryłowski L. (2019). Urokinase of plasminogen activator (uPA) in peritoneal fluid in patients with peritonitis. Pomeranian J. Life Sci..

[13] Robert-Boire V., Rosca L., Samson Y., Ospina L.H., Perreault S. (2017). Clinical Presentation and Outcome of Patients with Optic Pathway Glioma. Pediatr. Neurol..

[14] Jacob K., Quang-Khuong D.-A., Jones D.T.W., Witt H., Lambert S., Albrecht S., Witt O., Vezina C., Shirinian M., Faury D. (2011). Genetic Aberrations Leading to MAPK Pathway Activation Mediate Oncogene-Induced Senescence in Sporadic Pilocytic Astrocytomas. Clin. Cancer Res. Off. J. Am. Assoc. Cancer Res..

[15] Ryall S., Zapotocky M., Fukuoka K., Nobre L., Guerreiro Stucklin A., Bennett J., Siddaway R., Li C., Pajovic S., Arnoldo A. (2020). Integrated Molecular and Clinical Analysis of 1000 Pediatric Low-Grade Gliomas. Cancer Cell.

[16] Packer R.J., Pfister S., Bouffet E., Avery R., Bandopadhayay P., Bornhorst M., Bowers D.C., Ellison D., Fangusaro J., Foreman N. (2016). Pediatric Low-Grade Gliomas: Implications of the Biologic Era. Neuro-Oncology.

[17] Jones D.T.W., Kieran M.W., Bouffet E., Alexandrescu S., Bandopadhayay P., Bornhorst M., Ellison D., Fangusaro J., Fisher M.J., Foreman N. (2018). Pediatric Low-Grade Gliomas: Next Biologically Driven Steps. Neuro-Oncology.

[18] Huang M., Patel J., Patel B.C. Optic Nerve Glioma. https://pubmed.ncbi.nlm.nih.gov/32491801/.

[19] Azizi A.A., Walker D.A., Liu J.-F., Sehested A., Jaspan T., Pemp B., Simmons I., Ferner R., Grill J., Hargrave D. (2020). NF1 Optic Pathway Glioma: Analyzing Risk Factors for Visual Outcome and Indications to Treat. Neuro-Oncology.

[20] Northcott P.A., Pfister S.M., Jones D.T.W. (2015). Next-Generation (Epi)Genetic Drivers of Childhood Brain Tumours and the Outlook for Targeted Therapies. Lancet Oncol..

[21] Ostrom Q.T., de Blank P.M., Kruchko C., Petersen C.M., Liao P., Finlay J.L., Stearns D.S., Wolff J.E., Wolinsky Y., Letterio J.J. (2015). Alex’s Lemonade Stand Foundation Infant and Childhood Primary Brain and Central Nervous System Tumors Diagnosed in the United States in 2007–2011. Neuro-Oncology.

[22] Bandopadhayay P., Bergthold G., London W.B., Goumnerova L.C., Morales La Madrid A., Marcus K.J., Guo D., Ullrich N.J., Robison N.J., Chi S.N. (2014). Long-Term Outcome of 4040 Children Diagnosed with Pediatric Low-Grade Gliomas: An Analysis of the Surveillance Epidemiology and End Results (SEER) Database: PLGG SEER Long-Term Outcome. Pediatr. Blood Cancer.

[23] Krishnatry R., Zhukova N., Guerreiro Stucklin A.S., Pole J.D., Mistry M., Fried I., Ramaswamy V., Bartels U., Huang A., Laperriere N. (2016). Clinical and Treatment Factors Determining Long-Term Outcomes for Adult Survivors of Childhood Low-Grade Glioma: A Population-Based Study. Cancer.

[24] Colin C., Padovani L., Chappé C., Mercurio S., Scavarda D., Loundou A., Frassineti F., André N., Bouvier C., Korshunov A. (2013). Outcome Analysis of Childhood Pilocytic Astrocytomas: A Retrospective Study of 148 Cases at a Single Institution: Outcome Study of Pilocytic Astrocytomas. Neuropathol. Appl. Neurobiol..

[25] Penman C.L., Faulkner C., Lowis S.P., Kurian K.M. (2015). Current Understanding of BRAF Alterations in Diagnosis, Prognosis, and Therapeutic Targeting in Pediatric Low-Grade Gliomas. Front. Oncol..

[26] Geoerger B., Moertel C.L., Whitlock J., McCowage G.B., Kieran M.W., Broniscer A., Hargrave D.R., Hingorani P., Kilburn L.B., Mueller S. (2018). Phase 1 Trial of Trametinib Alone and in Combination with Dabrafenib in Children and Adolescents with Relapsed Solid Tumors or Neurofibromatosis Type 1 (NF1) Progressive Plexiform Neurofibromas (PN). J. Clin. Oncol..

[27] Ronsley R., Hounjet C.D., Cheng S., Rassekh S.R., Duncan W.J., Dunham C., Gardiner J., Ghag A., Ludemann J.P., Wensley D. (2021). Trametinib Therapy for Children with Neurofibromatosis Type 1 and Life-Threatening Plexiform Neurofibroma or Treatment-Refractory Low-Grade Glioma. Cancer Med..

[28] Manoharan N., Choi J., Chordas C., Zimmerman M.A., Scully J., Clymer J., Filbin M., Ullrich N.J., Bandopadhayay P., Chi S.N. (2020). Trametinib for the Treatment of Recurrent/Progressive Pediatric Low-Grade Glioma. J. Neurooncol..

[29] Jones D.T.W., Hutter B., Jäger N., Korshunov A., Kool M., Warnatz H.-J., Zichner T., Lambert S.R., Ryzhova M., Quang D.A.K. (2013). Recurrent Somatic Alterations of FGFR1 and NTRK2 in Pilocytic Astrocytoma. Nat. Genet..

[30] Sharma M.K., Zehnbauer B.A., Watson M.A., Gutmann D.H. (2005). RAS Pathway Activation and an Oncogenic RAS Mutation in Sporadic Pilocytic Astrocytoma. Neurology.

[31] Hata J., Barbour M., Huang M.A. (2021). LGG-02. Pediatric Low-Grade Glioma Risk Stratification in the Molecular Era. Neuro-Oncology.

[32] Miklja Z., Pasternak A., Stallard S., Nicolaides T., Kline-Nunnally C., Cole B., Beroukhim R., Bandopadhayay P., Chi S., Ramkissoon S.H. (2019). Molecular Profiling and Targeted Therapy in Pediatric Gliomas: Review and Consensus Recommendations. Neuro-Oncology.

[33] Khatua S., Gutmann D.H., Packer R.J. (2018). Neurofibromatosis Type 1 and Optic Pathway Glioma: Molecular Interplay and Therapeutic Insights. Pediatr. Blood Cancer.

[34] Sabbagh A., Pasmant E., Imbard A., Luscan A., Soares M., Blanché H., Laurendeau I., Ferkal S., Vidaud M., Pinson S. (2013). NF1 Molecular Characterization and Neurofibromatosis Type I Genotype-Phenotype Correlation: The French Experience. Hum. Mutat..

[35] Del Baldo G., Cacchione A., Dell’Anna V.A., Merli P., Colafati G.S., Marrazzo A., Rossi S., Giovannoni I., Barresi S., Deodati A. (2022). Rethinking the Management of Optic Pathway Gliomas: A Single Center Experience. Front. Surg..

[36] Cooney T., Yeo K.K., Kline C., Prados M., Haas-Kogan D., Chi S., Mueller S. (2019). Neuro-Oncology Practice Clinical Debate: Targeted Therapy vs Conventional Chemotherapy in Pediatric Low-Grade Glioma. Neuro-Oncol. Pract..

[37] Liu H., Chen Y., Qin X., Jin Z., Jiang Y., Wang Y. (2022). Epidemiology and Survival of Patients with Optic Pathway Gliomas: A Population-Based Analysis. Front. Oncol..

[38] Tabash M.A. (2019). Characteristics, Survival and Incidence Rates and Trends of Pilocytic Astrocytoma in Children in the United States; SEER-Based Analysis. J. Neurol. Sci..

[39] Knight J., De Jesus O. (2023). Pilocytic Astrocytoma.

[40] Collins V.P., Jones D.T.W., Giannini C. (2015). Pilocytic Astrocytoma: Pathology, Molecular Mechanisms and Markers. Acta Neuropathol..

[41] Chen Y.-H., Gutmann D.H. (2014). The Molecular and Cell Biology of Pediatric Low-Grade Gliomas. Oncogene.

[42] Campen C.J., Gutmann D.H. (2018). Optic Pathway Gliomas in Neurofibromatosis Type 1. J. Child Neurol..

[43] Peckham-Gregory E.C., Montenegro R.E., Stevenson D.A., Viskochil D.H., Scheurer M.E., Lupo P.J., Schiffman J.D. (2018). Evaluation of Racial Disparities in Pediatric Optic Pathway Glioma Incidence: Results from the Surveillance, Epidemiology, and End Results Program, 2000–2014. Cancer Epidemiol..

[44] Helfferich J., Nijmeijer R., Brouwer O.F., Boon M., Fock A., Hoving E.W., Meijer L., den Dunnen W.F.A., de Bont E.S.J.M. (2016). Neurofibromatosis Type 1 Associated Low Grade Gliomas: A Comparison with Sporadic Low Grade Gliomas. Crit. Rev. Oncol. Hematol..

[45] Blanchard G., Lafforgue M.-P., Lion-François L., Kemlin I., Rodriguez D., Castelnau P., Carneiro M., Meyer P., Rivier F., Barbarot S. (2016). Systematic MRI in NF1 Children under Six Years of Age for the Diagnosis of Optic Pathway Gliomas. Study and Outcome of a French Cohort. Eur. J. Paediatr. Neurol..

[46] Trevisson E., Cassina M., Opocher E., Vicenzi V., Lucchetta M., Parrozzani R., Miglionico G., Mardari R., Viscardi E., Midena E. (2017). Natural History of Optic Pathway Gliomas in a Cohort of Unselected Patients Affected by Neurofibromatosis 1. J. Neurooncol..

[47] Avery R.A., Mansoor A., Idrees R., Biggs E., Alsharid M.A., Packer R.J., Linguraru M.G. (2016). Quantitative MRI Criteria for Optic Pathway Enlargement in Neurofibromatosis Type 1. Neurology.

[48] Lassaletta A., Zapotocky M., Mistry M., Ramaswamy V., Honnorat M., Krishnatry R., Guerreiro Stucklin A., Zhukova N., Arnoldo A., Ryall S. (2017). Therapeutic and Prognostic Implications of BRAF V600E in Pediatric Low-Grade Gliomas. J. Clin. Oncol..

[49] Pagès M., Beccaria K., Boddaert N., Saffroy R., Besnard A., Castel D., Fina F., Barets D., Barret E., Lacroix L. (2017). Co-Occurrence of Histone H3 K27M and BRAF V600E Mutations in Paediatric Midline Grade I Ganglioglioma. Brain Pathol..

[50] Zhang K., Liu D., Yang Z. (2020). Prognostic role of BRAF mutation in low grade gliomas: A meta-analysis. World Neurosurg..

[51] Laurent D., Smith A.E., Bessler W.K., Mendonca M., Chin-Sinex H., Descovich M., Horvai A.E., Clapp D.W., Nakamura J.L. (2021). Irradiation of Nf1 mutant mouse models of spinal plexiform neurofibromas drives pathologic progression and decreases survival. Neurooncol. Adv..

[52] Lobón-Iglesias M.J., Laurendeau I., Guerrini-Rousseau L., Tauziède-Espariat A., Briand-Suleau A., Varlet P., Vidaud D., Vidaud M., Brugieres L., Grill J. (2020). NF1-like Optic Pathway Gliomas in Children: Clinical and Molecular Characterization of This Specific Presentation. Neuro-Oncol. Adv..

[53] Prada C.E., Hufnagel R.B., Hummel T.R., Lovell A.M., Hopkin R.J., Saal H.M., Schorry E.K. (2015). The Use of Magnetic Resonance Imaging Screening for Optic Pathway Gliomas in Children with Neurofibromatosis Type 1. J. Pediatr..

[54] Sellmer L., Farschtschi S., Marangoni M., Heran M.K.S., Birch P., Wenzel R., Mautner V.-F., Friedman J.M. (2018). Serial MRIs Provide Novel Insight into Natural History of Optic Pathway Gliomas in Patients with Neurofibromatosis 1. Orphanet J. Rare Dis..

[55] Diggs-Andrews K.A., Brown J.A., Gianino S.M., Rubin J.B., Wozniak D.F., Gutmann D.H. (2014). Sex Is a Major Determinant of Neuronal Dysfunction in Neurofibromatosis Type 1: Sex Impacts Outcomes in NF1. Ann. Neurol..

[56] Friedrich R.E., Nuding M.A. (2016). Optic Pathway Glioma and Cerebral Focal Abnormal Signal Intensity in Patients with Neurofibromatosis Type 1: Characteristics, Treatment Choices and Follow-up in 134 Affected Individuals and a Brief Review of the Literature. Anticancer. Res..

[57] Roonprapunt C., Abbott R. (2002). Surgical Treatment of Brainstem Gliomas in Children. Neurosurg. Q..

[58] Tsai J.W., Jungwhan J.C., Ouaalam H., Murillo E.A., Yeo K.K., Vogelzang J., Sousa C., Woods J.K., Ligon K.L., Warfield S.K. (2023). Integrated response analysis of pediatric low-grade gliomas during and after targeted therapy treatment. Neuro-Oncol. Adv..

[59] Ertiaei A., Hanaei S., Habibi Z., Moradi E., Nejat F. (2016). Optic Pathway Gliomas: Clinical Manifestation, Treatment, and Follow-Up. Pediatr. Neurosurg..

[60] Rasool N., Odel J.G., Kazim M. (2017). Optic Pathway Glioma of Childhood. Curr. Opin. Ophthalmol..

[61] Azizi A.A., Walker D.A., Liu J.-F., Sehested A., Jaspan T., Simmons I., Ferner R., Grill J., Hargrave D., Hernaiz-Driever P. (2018). NFM-04. Initial management strategy as a discriminator of visual outcome in children presenting with neurofibromatosis type 1 and optic pathway glioma—Results from a société internationale d’oncologie pédiatrique europe (siope) clinical trials workshop. Neuro-Oncology.

[62] Walker D.A., Azizi A.A., Liu J.-F., Sehested A., Jaspan T., Pemp B., Simmons I., Ferner R., Grill J., Hargrave D. (2020). Regarding “Neuro-Oncology Practice Clinical Debate: Targeted Therapy vs Conventional Chemotherapy in Pediatric Low-Grade Glioma”. Neuro-Oncol. Pract..

[63] Heidary G., Fisher M.J., Liu G.T., Ferner R.E., Gutmann D.H., Listernick R.H., Kapur K., Loguidice M., Ardern-Holmes S.L., Avery R.A. (2020). Visual Field Outcomes in Children Treated for Neurofibromatosis Type 1–Associated Optic Pathway Gliomas: A Multicenter Retrospective Study. J. Am. Assoc. Pediatr. Ophthalmol. Strabismus.

[64] Cassina M., Frizziero L., Opocher E., Parrozzani R., Sorrentino U., Viscardi E., Miglionico G., Midena E., Clementi M., Trevisson E. (2019). Optic Pathway Glioma in Type 1 Neurofibromatosis: Review of Its Pathogenesis, Diagnostic Assessment, and Treatment Recommendations. Cancers.

[65] Miller N.R. (2004). Primary Tumours of the Optic Nerve and Its Sheath. Eye.

[66] Zahavi A., Toledano H., Cohen R. (2018). Use of Optical Coherence Tomog- Raphy to Detect Retinal Nerve Fiber Loss in Children with Optic Pathway Glioma. Front. Neurol..

[67] Adhikari S., Dongol R.M., Hewett Y., Shah B.K. (2014). Vincristine-Induced Blindness: A Case Report and Review of Literature. Anticancer Res..

[68] Parrozzani R., Miglionico G., Leonardi F., Pulze S., Trevisson E., Clementi M., Opocher E., Licata V., Viscardi E., Pilotto E. (2018). Correlation of Peripapillary Retinal Nerve Fibre Layer Thickness with Visual Acuity in Paediatric Patients Affected by Optic Pathway Glioma. Acta Ophthalmol..

[69] Yanni S.E., Wang J., Cheng C.S., Locke K.I., Wen Y., Birch D.G., Birch E.E. (2013). Normative Reference Ranges for the Retinal Nerve Fiber Layer, Macula, and Retinal Layer Thicknesses in Children. Am. J. Ophthalmol..

[70] Parrozzani R., Clementi M., Kotsafti O., Miglionico G., Trevisson E., Orlando G., Pilotto E., Midena E. (2013). Optical Coherence Tomography in the Diagnosis of Optic Pathway Gliomas. Investig. Ophthalmol. Vis. Sci..

[71] Avery R.A., Mansoor A., Idrees R., Trimboli-Heidler C., Ishikawa H., Packer R.J., Linguraru M.G. (2016). Optic Pathway Glioma Volume Predicts Retinal Axon Degeneration in Neurofibromatosis Type 1. Neurology.

[72] Listernick R., Ferner R.E., Liu G.T., Gutmann D.H. (2007). Optic Pathway Gliomas in Neurofibromatosis-1: Controversies and Recommendations. Ann. Neurol..

[73] Avery R.A., Cnaan A., Schuman J.S., Trimboli-Heidler C., Chen C.-L., Packer R.J., Ishikawa H. (2015). Longitudinal Change of Circumpapillary Retinal Nerve Fiber Layer Thickness in Children with Optic Pathway Gliomas. Am. J. Ophthalmol..

[74] Parrozzani R., Clementi M., Frizziero L., Miglionico G., Perrini P., Cavarzeran F., Kotsafti O., Comacchio F., Trevisson E., Convento E. (2015). In Vivo Detection of Choroidal Abnormalities Related to NF1: Feasibility and Comparison with Standard NIH Diagnostic Criteria in Pediatric Patients. Investig. Ophthalmol. Vis. Sci..

[75] Alpfidan I., Sakarya Y., Goktas S., Ozcimen M., Sakarya R. (2014). Ophthalmological Assessment of Children with Neurofibromatosis Type 1. Eur. J. Pediatr..

[76] Parrozzani R., Pilotto E., Clementi M., Frizziero L., Leonardi F., Convento E., Miglionico G., Pulze S., Perrini P., Trevisson E. (2018). Retinal Vascular Abnormalities in a Large Cohort of Patients Affected by Neurofibromatosis Type 1: A Study Using Optical Coherence Tomography Angiography. Retina.

[77] Aridgides P., Janssens G.O., Braunstein S., Campbell S., Poppe M., Murphy E., MacDonald S., Ladra M., Alapetite C., Haas-Kogan D. (2020). Gliomas, Germ Cell Tumors, and Craniopharyngioma. Pediatr. Blood Cancer.

[78] de Lucio Delgado A., Villegas Rubio J.A., Riaño-Galán I., Pérez Gordón J. (2023). Effect of the Use of Gnrh Analogs in Low-Grade Cerebral Glioma. Children.

[79] Śledzińska P., Bebyn M., Szczerba E., Furtak J., Harat M., Olszewska N., Kamińska K., Kowalewski J., Lewandowska M.A. (2022). Glioma 2021 WHO Classification: The Superiority of NGS Over IHC in Routine Diagnostics. Mol. Diagn. Ther..

[80] Kim H., Zheng S., Amini S.S., Virk S.M., Mikkelsen T., Brat D.J., Grimsby J., Sougnez C., Muller F., Hu J. (2015). Whole-genome and multisector exome sequencing of primary and post-treatment glioblastoma reveals patterns of tumor evolution. Genome Res..

[81] Campbell A.A., Gartrell-Corrado R.D., Mansukhani M., Zanazzi G., Canoll P., Garvin J.H., Kazim M. (2020). SETD2 Mutation in an Aggressive Optic Nerve Glioma. JAMA Ophthalmol..

[82] de Blank P.M.K., Fisher M.J., Liu G.T., Gutmann D.H., Listernick R., Ferner R.E., Avery R.A. (2017). Optic Pathway Gliomas in Neurofibromatosis Type 1: An Update: Surveillance, Treatment Indications, and Biomarkers of Vision. J. Neuroophthalmol..

[83] Avery R.A., Bouffet E., Packer R.J., Reginald A. (2013). Feasibility and Comparison of Visual Acuity Testing Methods in Children with Neurofibromatosis Type 1 and/or Optic Pathway Gliomas. Investig. Ophthalmol. Vis. Sci..

[84] Kelly J.P., Weiss A.H. (2013). Detection of Tumor Progression in Optic Pathway Glioma with and without Neurofibromatosis Type 1. Neuro-Oncology.

[85] Cassiman C., Laenen A., Jacobs S., Demaerel P., Legius E., Casteels I. (2018). Ophthalmological Examination in Neurofibromatosis Type 1: A Long-Term Retrospective Analysis. Acta Ophthalmol..

[86] Patel D.E., Cumberland P.M., Walters B.C., Russell-Eggitt I., Cortina-Borja M., Rahi J.S., OPTIC Study Group (2015). Study of Optimal Perimetric Testing In Children (OPTIC): Normative Visual Field Values in Children. Ophthalmology.

[87] Fried I., Tabori U., Tihan T., Reginald A., Bouffet E. (2013). Optic Pathway Gliomas: A Review. CNS Oncol..

[88] Gnekow A.K., Kandels D., van Tilburg C., Azizi A.A., Opocher E., Stokland T., Driever P.H., Meeteren A.Y.N.S., Thomale U.W., Schuhmann M.U. (2019). SIOP-E-BTG and GPOH guidelines for diagnosis and treatment of children and adolescents with low grade glioma. Klin. Padiatr..

[89] Binning M.J., Liu J.K., Kestle J.R.W., Brockmeyer D.L., Walker M.L. (2007). Optic pathway gliomas: A review. Neurosurg. Focus.

[90] Ater J.L., Zhou T., Holmes E., Mazewski C.M., Booth T.N., Freyer D.R., Lazarus K.H., Packer R.J., Prados M., Sposto R. (2012). Randomized Study of Two Chemotherapy Regimens for Treatment of Low-Grade Glioma in Young Children: A Report from the Children’s Oncology Group. J. Clin. Oncol..

[91] Lassaletta A., Scheinemann K., Zelcer S.M., Hukin J., Wilson B.A., Jabado N., Carret A.S., Lafay-Cousin L., Larouche V., Hawkins C.E. (2016). Phase II Weekly Vinblastine for Chemotherapy-Naïve Children with Progressive Low-Grade Glioma: A Canadian Pediatric Brain Tumor Consortium Study. J. Clin. Oncol..

[92] Dodgshun A.J., Maixner W.J., Heath J.A., Sullivan M.J., Hansford J.R. (2016). Single Agent Carboplatin for Pediatric Low-Grade Glioma: A Retrospective Analysis Shows Equivalent Efficacy to Multiagent Chemotherapy: Carboplatin in Pediatric Low Grade Glioma. Int. J. Cancer.

[93] Zhukova N., Rajagopal R., Lam A., Coleman L., Shipman P., Walwyn T., Williams M., Sullivan M., Campbell M., Bhatia K. (2018). Use of bevacizumab as a single agent or in adjunct with traditional chemotherapy regimens in children with unresectable or progressive low-grade glioma. Cancer Med..

[94] Presta L.G., Chen H., O’Connor S.J., Chisholm V., Meng Y.G., Krummen L., Winkler M., Ferrara N. (1997). Humanization of an Anti-Vascular Endothelial Growth Factor Monoclonal Antibody for the Therapy of Solid Tumors and Other Disorders. Cancer Res..

[95] Fangusaro J., Gururangan S., Poussaint T.Y., McLendon R.E., Onar-Thomas A., Warren K.E., Wu S., Packer R.J., Banerjee A., Gilbertson R.J. (2013). Bevacizumab (BVZ)-Associated Toxicities in Children with Recurrent Central Nervous System Tumors Treated with BVZ and Irinotecan (CPT-11). Cancer.

[96] Avery R.A., Hwang E.I., Jakacki R.I., Packer R.J. (2014). Marked Recovery of Vision in Children with Optic Pathway Gliomas Treated with Bevacizumab. JAMA Ophthalmol..

[97] Gorsi H., Khanna P.C., Tumblin M., Yeh-Nayre L., Milburn M., Elster J., Crawford J.R. (2018). Single-Agent Bevacizumab in the Treatment of Recurrent or Refractory Pediatric Low-Grade Glioma: A Single Institutional Experience. Pediatr. Blood Cancer.

[98] Gururangan S., Fangusaro J., Poussaint T.Y., McLendon R.E., Onar-Thomas A., Wu S., Packer R.J., Banerjee A., Gilbertson R.J., Fahey F. (2013). Efficacy of Bevacizumab plus Irinotecan in Children with Recurrent Low-Grade Gliomas—A Pediatric Brain Tumor Consortium Study. Neuro-Oncology.

[99] Heng M.A., Padovani L., Dory-Lautrec P., Gentet J.C., Verschuur A., Pasquier E., Figarella-Branger D., Scavarda D., André N. (2016). Can Metronomic Maintenance with Weekly Vinblastine Prevent Early Relapse/Progression after Bevacizumab–Irinotecan in Children with Low-Grade Glioma?. Cancer Med..

[100] Packer R.J., Jakacki R.I., Horn M., Rood B., Vezina G., MacDonald T.J., Fisher M.J., Cohen B.H. (2009). Objective Response of Multiply Recurrent Low-Grade Gliomas to Bevacizumab and Irinotecan. Pediatr. Blood Cancer.

[101] Couec M.-L., André N., Thebaud E., Minckes O., Rialland X., Corradini N., Aerts I., Bérard P.M., Bourdeaut F., Leblond P. (2012). Bevacizumab and Irinotecan in Children with Recurrent or Refractory Brain Tumors: Toxicity and Efficacy Trends. Pediatr. Blood Cancer.

[102] Kalra M., Heath J.A., Kellie S.J., Pozza L.D., Stevens M., Swamy S., McCowage G. (2015). Confirmation of Bevacizumab Activity, and Maintenance of Efficacy in Retreatment after Subsequent Relapse, in Pediatric Low-Grade Glioma. J. Pediatr. Hematol. Oncol..

[103] Louis D.N., Perry A., Wesseling P., Brat D.J., Cree I.A., Figarella-Branger D., Hawkins C., Ng H.K., Pfister S.M., Reifenberger G. (2021). The 2021 WHO Classification of Tumors of the Central Nervous System: A Summary. Neuro-Oncology.

[104] Green K., Panagopoulou P., D’Arco F., O’Hare P., Bowman R., Walters B., Dahl C., Jorgensen M., Patel P., Slater O. (2023). A Nationwide Evaluation of Bevacizumab-Based Treatments in Pediatric Low-Grade Glioma in the UK: Safety, Efficacy, Visual Morbidity, and Outcomes. Neuro-Oncology.

[105] Tsang D.S., Murphy E.S., Merchant T.E. (2017). Radiation Therapy for Optic Pathway and Hypothalamic Low-Grade Gliomas in Children. Int. J. Radiat. Oncol..

[106] Selt F., van Tilburg C.M., Bison B., Sievers P., Harting I., Ecker J., Pajtler K.W., Sahm F., Bahr A., Simon M. (2020). Response to Trametinib Treatment in Progressive Pediatric Low-Grade Glioma Patients. J. Neuro-Oncol..

[107] Perreault S., Larouche V., Tabori U., Hawkin C., Lippé S., Ellezam B., Décarie J.-C., Théoret Y., Métras M.-É., Sultan S. (2019). A Phase 2 Study of Trametinib for Patients with Pediatric Glioma or Plexiform Neurofibroma with Refractory Tumor and Activation of the MAPK/ERK Pathway: TRAM-01. BMC Cancer.

